# 55564 Interactive mindfulness and dialogue sessions are integral components of research training.

**DOI:** 10.1017/cts.2021.569

**Published:** 2021-03-30

**Authors:** Kit Knier, Adriana Morales Gomez, Joanna Yang Yowler, Chris Pierret, Linda M. Scholl

**Affiliations:** 1 Mayo Clinic Graduate School of Biomedical Sciences, Mayo Clinic, Rochester, MN, USA; 2 Mayo Clinic Medical Scientist Training Program, Mayo Clinic, Rochester, MN, USA; 3 Department of Research, Mayo Clinic, Jacksonville, FL, USA; 4 Department of Biochemistry and Molecular Biology, Mayo Clinic, Rochester, MN, USA; 5 Office of Applied Scholarship and Education Science, Mayo Clinic College of Medicine, Mayo Clinic, Rochester, MN, USA

## Abstract

ABSTRACT IMPACT: This work demonstrates the integration of interactive mindfulness and dialogue sessions in curricula is both desired by students and effective in conferring resilience, a protective factor that may aid in maintaining wellbeing of trainees interested in pursuing graduate studies in biomedical research and science. OBJECTIVES/GOALS: To support student futures in the field of biomedicine, Mayo Clinic Graduate School of Biological Sciences utilized digital platforms to deliver a summer research program in the summer of 2020. One goal of this program, in addition to scholastic outcomes and research experience, was to support and improve the wellbeing of college student participants. METHODS/STUDY POPULATION: Following the cancellation of in-person summer research programs, students were invited to attend a digital Summer Foundations in Research program. The 4-week program included 4 small group dialogue sessions led by trained facilitators and 4 large group mindfulness seminars followed with 3 Q/A style small group sessions. Surveys were delivered on days 1, 27, and 3 months following the program. Wellbeing measures included Brief Resilience, Perceived Stress, and Satisfaction with Life Scales. Students were prompted to indicate how worthwhile they found course components and comment on why they rated each component the way they did. Wellbeing results were assessed using paired t-tests with Bonferroni correction for multiple comparisons. Thematic analysis was used to interpret qualitative results. RESULTS/ANTICIPATED RESULTS: Students improved across all wellbeing measures at the program conclusion, including resilience (mean difference(SE) pre- to post-program +0.22(0.06) p=0.0007), perceived stress (-1.71(0.66) p=0.0116), and life satisfaction (+1.57(0.52) p=0.0037). Gains in resilience were maintained 3 months out (pre-program to 3 month survey +0.28(0.06) p<0.0001). To our surprise, mindfulness was the highest rated component of the research program with 85% (121/142) of students rating the mindfulness component ‘extremely’ or ‘quite worthwhile.’ At 3 months, 81% (74/91) reported continued use of one or more skills learned in the mindfulness sessions. Student comments endorsed the perceived importance of interactive mindfulness and dialogue sessions to the program and to careers in biomedical science and research. DISCUSSION/SIGNIFICANCE OF FINDINGS: Our results support the use of interactive mindfulness and dialogue programming as a participant supported, evidence-based approach to strengthen the resilience of undergraduate students pursuing careers in biomedicine. In the future, booster programming may be considered to maintain improvements in perceived stress and life satisfaction.

